# Trastuzumab Charge Variants: a Study on Physicochemical and Pharmacokinetic Properties

**DOI:** 10.52547/ibj.3837

**Published:** 2023-01-01

**Authors:** Fatemeh Torkashvand, Mahsa Mehranfar, Mahsa Rashidi Gero, Parisa Jafarian, Esmat Mirabzadeh, Bahareh Azarian, Soroush Sardari, Behrouz Vaziri

**Affiliations:** 1 Biotechnology Research Centre, Pasteur Institute of Iran, Tehran, Iran;; 2 Department of Biology, Science and Research Branch, Islamic Azad University, Tehran, Iran;; 3 Department of Biochemistry, Faculty of Biological Sciences, North Tehran branch, Islamic Azad University, Tehran, Iran; ¶ These authors contributed equally to this work.

**Keywords:** Monoclonal antibodies, Pharmacokinetics, Trastuzumab

## Abstract

**Background:**

Post-translational modifications in bioprocessing and storage of recombinant mAbs are the main sources of charge variants. While the profile of these kinds of variants is considered an important quality attribute of therapeutic mAbs, there is controversy about their direct role on safety and efficacy. In this study, the physicochemical and PK properties of separated charge variants belonging to a trastuzumab potential biosimilar were examined.

**Methods:**

The APs, BPs, and main variants of trastuzumab were separated and enriched by semi-preparative WCX. A panel of analytical techniques was utilized to characterize the physicochemical properties of these variants. The binding affinity to HER2 and FcγRs and the PK parameters were evaluated for each variant.

**Results:**

Based on the results, the charge variants of the proposed biosimilar had no significant influence on the examined efficacy and PK parameters.

**Conclusion:**

During the development and production of biosimilar mAbs, studying the effects of their charge variants on efficacy and PK parameters is needed.

## Introduction

Developments in the bioprocessing of mAbs have drawn attention to the characterization of their heterogeneous variants^[^^1^^,^^2^^]^. Typically, mAbs undergo enzymatic and chemical changes during cell culture expression, downstream process, formulation, and shelf-life^[^^3^^,^^4^^]^, which might modify their size, hydrophobicity, glycosylation pattern, and particularly their surface charge properties^[^^5^^]^. Charge species comprise several modifications influencing the overall surface charge and the p*I* value of the mAbs^[^^6^^,^^7^^]^. For regulatory authorities, consistency in the charge variants profile, i.e. similarity in quantity and pattern of a biotherapeutic is considered a quality requirement. 

Analytical methods such as ion exchange chromatography or isoelectric focusing IEF have been used to determine charge variants^[^^2^^,^^8^^]^. There are contradictory data on the role of these variants in mAbs efficacy, PK, and effector function mediated by FcγRs^[^^9^^-^^11^^]^. Some reports have shown that charge variants have similar FcRn binding affinity, PK behavior, and *in vitro* potency like the main peak of an IgG1 product^[^^12^^]^ and a biosimilar product of Avastin^[^^13^^]^. It has also been demonstrated that charge variants with one or less than one unit in p*I* value showed no significant difference in terms of serum half-life^[^^14^^-^^16^^]^. On the other hand, one report revealed that charge variants significantly affected safety and efficacy^[^^11^^]^.

In the current study, the efficacy and PK of a proposed biosimilar of trastuzumab charge variant were investigated. The acidic, basic and main variants of trastuzumab were separated by WCX. The separated variants underwent physicochemical characterization via various orthogonal analytical techniques, including analytical WCX chromatography, cIEF, CE-SDS, 2-DE, SEC, and CD. SPR method was used to study the affinity of trastuzumab charge variants to HER2 and FcγRs. The SPR analysis to examine the binding affinity of charge variants to FcRn was performed as an *in vitro *PK assessment. Measurement of blood clearance of trastuzumab in rats was also carried out using ELIZA as an *in vivo* PK assessment.

## MATERIALS AND METHODS

**Materials**

Trastuzumab biosimilar was kindly gifted by AryoGen Pharmed Co. (Alborz, Iran). Chemicals were purchased from Merck (Germany). L-histidine HCl, L-histidine, polysorbate 20, and trehalose were provided by Sigma-Aldrich (Saint Louis, MO). Acrylamide, N’,N’,N’ Bis-acrylamide, ammonium persulfate, and tetramethylethylenediamine were bought from Bio-Rad (USA). 

**Separation of charge variants**

A method previously introduced by Griaud et al.^[^^17^^]^ was used to fractionate the charge variants. Briefly, a gradient of increasing concentration of NaCl (from 5.5% to 13.5% in 25 mM of Tris, pH 7.8) was applied to a PROPAC 10-WCX Semi-Preparative column (9 × 250 mm; Thermo Fisher Scientific, USA, 063474) with 3 mL.min^-1^ flow rate for 75 min using a KNAUER AZURA HPLC system and a UV detection of 218 nm. Fractions with more than 90% purity were then pooled. The buffer exchange of separated charge variant fractions was performed by Amicon Stirred Ultrafiltration Cell (Millipore^®^, USA) using a membrane with 10 kDa cut-off. The isolated fraction concentration was measured at 280 nm with an extinction coefficient of 1.49 mL. (mg cm)^-1^, then their concentrations adjusted to 10 mg.mL^-1^ and sterilized by a 0.22-µm syringe filter. 

**Analytical cation exchange chromatography**

PROPAC 10-WCX analytical column (4 × 250 mm; Thermo Fisher Scientific, 054993) was applied to measure the purity of the fractions with the method mentioned in the section “separation of charge variants”, except for flow rate and the injection amount, which were 1.0 mL.min-1 and 50 µg, respectively. 

**Capillary isoelectric focusing**

cIEF was carried out using 50 μm internal diameter × 45 cm neutral coated capillary (PN 477441, Beckman Coulter, USA) with an effective length of 20 cm. An Agilent 1600 CE instrument (Germany) with detector filter assembly (PN G1600-62700) was used. Samples were desalted and concentrated to 5 mg.mL^-1^. Then 200 µL of the cIEF gel solution (PN 477497, Beckman Coulter), 12 µL of pH 3-10 Pharmalyte®, 20 µL of cathodic stabilizer (500 Mm of L-arginine), 2 µL of anodic stabilizer (200 mM of iminodiacetic acid), and 2 µL of the p*I* markers 5.5, 7, and 9.5 (PN A58481a, Beckman Coulter) were added to the samples prior to analysis. The detection wavelength was recorded at 280 nm. Critical parameters were chosen for method development according to the technique described previously^[^^18^^]^. 

**Two-dimensional electrophoresis**

Immobilized pH gradient strips (17 cm; pH 7-10; BioRad, USA) was rehydrated in 7 M of urea, 2 M of thiourea, 2% carrier ampholytes (pH 7-10), 70 mM of dithiothreitol, 0.001% bromophenol blue, and 100 µg of sample for 16 h. IEF was performed by PROTEAN IEF cell (BioRad) at 10,000 V and 20 °C (50 µA/strip for 50,000 Vh). Immobilized pH gradient strips were treated with a buffer containing 50 mM of Tris–HCl (pH 8.8), 6 M of urea, 20% glycerol, 2% sodium dodecyl sulfate, 0.01% bromophenol blue, and 2% DTT; the strips were then alkylated for another 15 min in the same buffer containing 2.5% iodoacetamide instead of DTT. They were loaded on 12% SDS-PAGE, applied 16 mA/gel for 30 min and 24 mA/gel for 5 h at 20 °C and stained by colloidal Coomassie Blue^[^^19^^]^.

**Capillary gel electrophoresis**

The reducing and non-reducing CGE were used for the analysis of the mAb charge variant fraction. The capillary electrophoresis instrument was Agilent 1600 (Germany). The internal diameter of a bare fused silica capillary with internal diameter of 50 μm and 30.2 cm with 20 cm effective length was used. Using an IgG purity/heterogeneity assay kit (PN A10663, Beckman Coulter), 100 µg of antibody solution was mixed with SDS sample buffer. The non-reduced samples were treated with 5 µL of iodoacetamide (250 mM), and the reduced samples were treated with 5 µL of 2-ME. Then 2 µL of the 10-kDa protein standard was added to each sample. The method was carried out according to the application note^[^^20^^]^, which applied -500 V/cm during 30 min for the reduced and 40 min for the non-reduced samples.

**Size exclusion chromatography-high-performance liquid chromatoghraphy**

Molecular weight related impurities were detected by SEC using a TSK-Gel G3000 column (Tosoh Bioscience, Tokyo, Japan; 5 mm, 7.8 mm × 300 mm) and the KNAUER AZURA HPLC system (Germany) at ambient temperature. At the end, 100 µg of the samples was eluted on the column over 30 minutes and detected at 280 nm. 

**HER2 binding kinetics**

Anti-human IgG (Fc) antibody (GE Healthcare, USA) was diluted in 10 mM of sodium acetate buffer, pH 5.0, and immobilized on CM_5_ chip as described in the Human Antibody Capture Kit (GE Healthcare)^[^^21^^]^. Each isolated charge variant (as a ligand) was diluted with the HBS-EP buffer (10 mM of HEPES [pH 7.4], 150 mM of NaCl, 50 μM of EDTA, and 0.005% [v/v] P20) and loaded on the chip. A single-cycle kinetic procedure was applied, and five different concentrations of HER2 extracellular domain (8.6 nM to 138 nM; Sigma- Aldrich) were passed over the chip. The association and dissociation times were 4 min and 9 min, respectively. A 30 s injection of the regeneration solution (3 M of magnesium chloride; pH 3.0) was used to regenerate the chip surface at the end of the runs. The data were analyzed by Biacore-X100 Evaluation software.

**FcγRIIIa and FcγRIIIb binding kinetics**

FcγRIIIa (CD16a) and FcγRIIIb (CD16b) binding kinetics were analyzed using SPR via a BIAcore X100 instrument (GE Healthcare). Anti His-tag antibodies were immobilized on CM_5_ chip (GE Healthcare) according to the His Capture Kit (GE Healthcare) manual^[^^22^^]^. FcγRIIIa and FcγRIIIb (Sino Biologicals, USA) were diluted with the running buffer (described in the section “HER2 binding kinetics”) to reach the concentration of 20 µg.mL^-1^ and then loaded on separated chips as ligands. Afterwards, different concentrations of each isolated charge variant of the mAb (analyte)( 2.5 µM to 40 µM) were passed over each chip via a single-cycle approach. The chip surface was regenerated by 10 mM of glycine-HCl buffer, pH 1.5. Data analysis was performed by Biacore-X100 Evaluation software.

**FcRn binding kinetics**

A BIAcore X100 SPR biosensor (GE Healthcare) was used to study the interaction of soluble FcRn with the isolated charge variants of the mAbs. Recombinant human FcRn (5 µg.mL^-1^ in 10 mM of sodium acetate; pH 5.0) was immobilized onto a CM_5_ biosensor chip via Amine Coupling Kit (GE Healthcare). The target mAb samples were diluted in PBS/P20 (50 mM of sodium phosphate [pH 6.0], 150 mM of NaCl, 0.02% NaN3, and 0.01% P20), used as running buffer in equilibrium binding experiments. A range of mAb concentrations from 0.41 to 6.65 µM was used with association and dissociation times of 180 seconds. The sensor chip surface was regenerated by a short injection of PBS/0.05% P20 at pH 8.0. The data were analyzed by Biacore-X100 Evaluation software.

**CD analysis**

Far-UV CD was employed to detect whether any substantial modification in the secondary structure of the charge variants. Secondary structure analysis was recorded using the Jasco J-810 spectrophotometer (Japan) at ambient temperature. The spectra were recorded at a wavelength ranging between 190 and 250 nm by a 2 nm step size and a 1 nm bandwidth in a cell with a path length of 1 mm and a scanning speed of 500 nm.min^-1^. To achieve a concentration of 0.5 mg.mL^-1^, the buffer of the mAb charge variants was exchanged four times with 10 mM of potassium phosphate buffer. The spectra were corrected and analyzed in duplicate, and the data were recorded by the Spectra Manager for Windows version 1.53.02. 

**PK study design and sampling**

The Department of Laboratory Animals at Pasteur Institute of Iran (Tehran) provided 250 g weighted male *Rattus norvegicus* aged 12–14 weeks. The animals were adapted for seven days in a group condition prior to receiving the injection. They had standard feeding and were maintained at room temperature with relative humidity of 50-55% and 12 h intervals of light/dark cycles. Healthy male Wistar rats were randomly allocated into three different groups (n=3), namely acidic, basic and main groups to cover 11 serum collection time points (pre-dose, 0.08, 2, 4, 8, 24, 48, and 168 [7 days], 336 [14 days], 504 [21 days], and 672 [28 days] hours post-dose injection). 10 mg.kg^-1^ of each charge variant was intravenously injected through the lateral tail vein. About 300 µl of the blood samples were collected via tail snip. Prior to collection of the blood, local anesthesia (spray of 10% lidocaine) was applied on the tail. Following 10 minutes, the tail was wiped clean with alcohol, and a 3-4 mm cut was made from the tip of the tail using sterile sharp scissors. For a suitable blood flow, a hot water bag (40 °C) was placed on the base of the tail, and then blood was collected by removing the tail scab. One hour incubation at room temperature was followed by centrifugation at 9300 ×g for 7 min to separate the samples. After the final blood collection, all the rats were anesthetized with ketamine (70 mg.kg^-1^) and xylazine (5 mg.kg^-1^) and euthanized by carbon dioxide. Finally, the corpses were disposed in an appropriate and standard way. The ELISA using a Trastuzumab (Herceptin®) Pharmacokinetic ELISA kit (Bioscience, Netherlands, Catalog MBS378011) was the method of choice for quantifying mAb levels in the serum samples at different time points. Standard samples and diluted serum samples were assayed in duplicate, whereas a log-log standard curve was constructed by Gen 5 software with a four-parameter fit to acquire sample concentration. Time-concentration plots were created using non-compartmental analysis (Phoenix WinNonLin V 6.3 software, Mountain View, CA, USA). PK parameters such as the maximum serum concentration (C_max_), the elimination half-life (t^1^/_2_), and the area under curve from 0 to 28 days (AUC_0-28d_) were assessed by the above-mentioned software. Statistical analysis was performed with SPSS software and one-way ANOVA. The data with *p* ˂ 0.05 were considered as statistically significant.

**Fig. 1:**
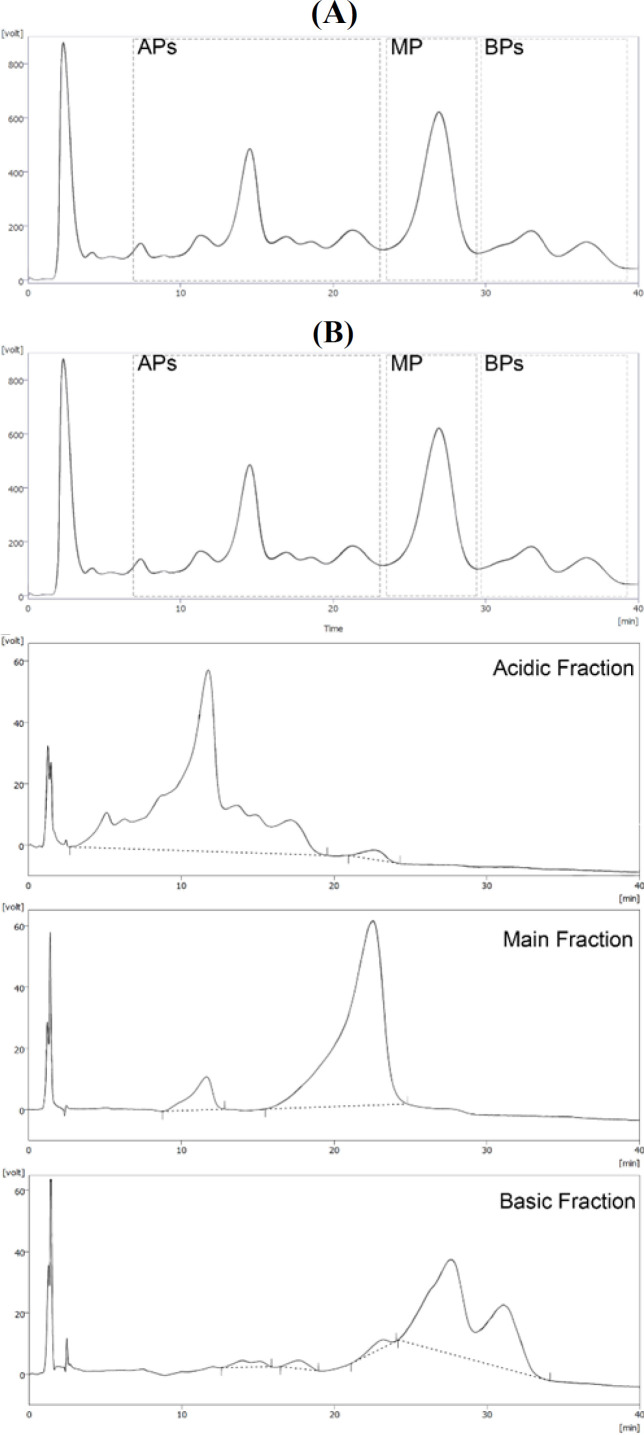
CEX of the trastuzumab and its collected fractions. (A) Semi-preparative CEX chromatogram of trastuzumab. The dashed boxes represent the selected pattern for fractionation; (B) analytical CEX chromatograms of charge variant fractions. The purity of the collected samples was calculated by manual integration (dashed baseline) of peaks in each chromatogram

## RESULTS

**Charge variant separation and physicochemical characterization**

**Fig. 2.**
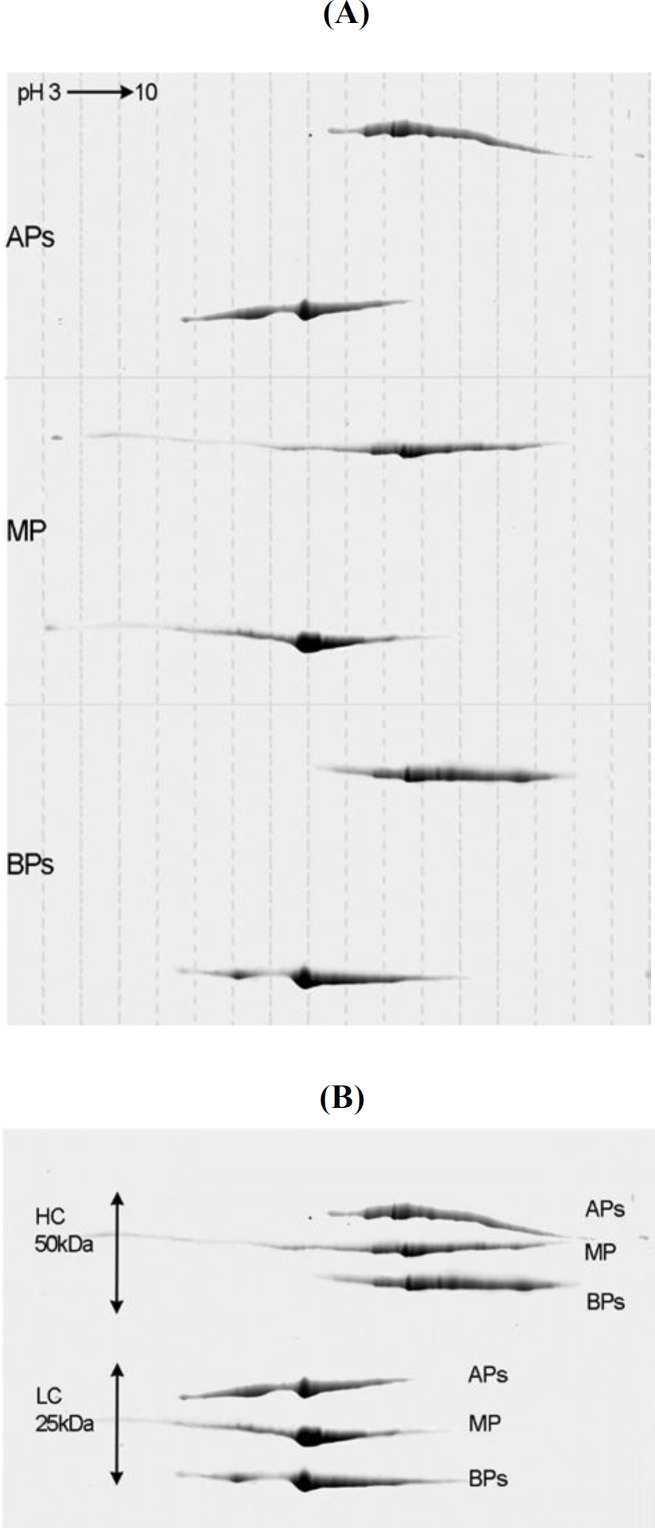
Two-dimensional electrophoresis analysis of (A) acidic (APs), main (MP), and basic variants (BPs) of trastuzumab under reducing conditions; (B) Combined image of heavy and light chains of different variants

Charge variants were separated from mAb using the semi-preparative WCX chromatography, and the fractions were collected as APs, MP, and BPs (Fig. 1A). Analytical CEX was used to evaluate the purity of fractions (Fig. 1B and Table 1). Furthermore, cIEF was used to characterize these fractions based on their p*I*^[^^23^^]^. In order to construct the p*I*-migration time plot, four p*I* markers were co-injected with the trastuzumab. The results displayed a narrow p*I* range (<1 units) with different peak densities. The calculated p*I* values of the APs, and BPs varied between 8.1 -8.4, and 8.6 - 8.7, respectively. *pI* value of the MP was 8.5. The coefficient of variation was less than 0.05 for three replicates (Supplementary Fig. 1A). Obviously, the cIEF pattern of charge distribution in each collected fraction was highly similar to their CEX charge distribution pattern (Supplementary Fig. 1B). Based on 2-DE, acidic diversity likely occurred in the light chain, while basic diversity happened in the heavy chain (Fig. 2). Evaluation of the secondary structure of the charge variants via CD analysis (190-250 nm) showed the comparable CD spectra in the region between 195 and 250 nm, intersecting the zero line at 200 nm and their negative extremes at approximately 214-217 nm (Supplementary Fig. 2). Some slight changes were observed in the CD spectra around 190-195 nm, and the positive extremes of the samples were shifted. It is evident that the MP had a proportionally negative amplitude at 190 nm. According to the secondary structure data analysis (Supplementary Table 1), the β- sheet structure had a higher percentage in the MP variant. Furthermore, the three variants were much similar in alpha, turn, and coil structures. Comparison of trastuzumab SEC chromatograms and the separated charge variants revealed a very similar size distribution among the samples having more than 99% of monomer peak with no significant difference in amount of the molecular weight-dependent impurities (Supplementary Fig. 3). The amount of non-glycosylated heavy chain was measured by reducing CGE (Supplementary Fig. 4). The non-glycosylated heavy chain approximately comprised 1%, 0.9%, and 0.7% of the main form, acidic variants, and basic variants of trastuzumab, respectively.

**Table 1:** The relative abundance of trastuzumab charge variants analyzed by semi-preparative CEX, and the purity of the isolated charge variants measured by analytical CEX

Variant	Relative abundance (semi-preparative CEX) (%)	Purity of isolated variants (analytical CEX) (%)
Acidic	37.7	98.1
Main	43.4	90.1
Basic	18.9	92.1

**Charge variant functional analysis**

***HER2, FcRIIIa, FcRIIIb binding kinetics***

The trastuzumab charge variant affinity to HER2, FcRIIIa, and FcRIIIb receptors was determined by the SPR method **(**Supplementary Table 2**).** The SPR analysis showed that the binding affinity (KD) of APs, MP, and BPs towards HER2 was similar, and their KD values were 3.59 × 10^-9^, 2.03 × 10^-9^, and 2.78 × 10^-9^ M, respectively. As shown in Supplementary Table 2, the binding affinity of charge variants towards FcRIIIa and FcRIIIb was also comparable. The results demonstrated that FcRIIIa binds to the acidic, main and basic fractions with KD of 2.65 × 10^-6^, 2.54 × 10^-6^, and 3.34 × 10^-6^ M, respectively. The amount of KD for FcRIIIb towards APs, MP, and BPs was 1.45 × 10^-5^, 1.09 × 10^-5^, and 1.29 × 10^-5 ^M, respectively.

***FcRn binding kinetics (in vitro PK study)***

The Fc-FcRn affinity analysis of the separated charge variants belonging to mAb was evaluated by the SPR method **(**Table 2**). **The KD of the acidic and basic variants of trastuzumab was slightly more than that of the main variant of the mAb. This amount of difference caused no significant difference in *in vivo* PK parameters (Table 2).

***In vivo PK study in rats***

**Fig. 3:**
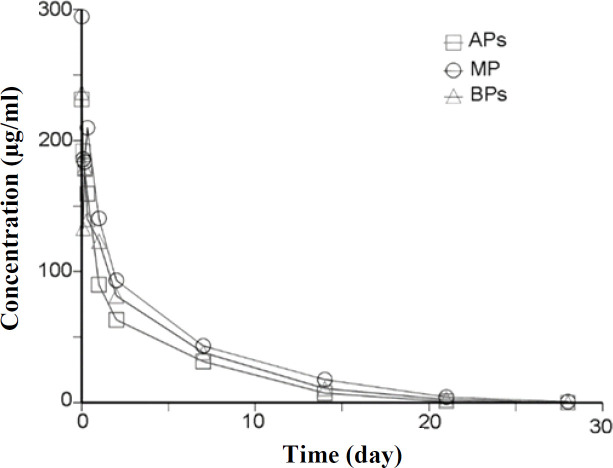
Serum concentration versus the time plot of main, acidic, and basic variants following IV administration of 10 mg.kg^-1^ of trastuzumab variants in male rats within 28 days

The variants showed similar t_1/2_, AUC_0-28d_, C_max_, and C_min_ values (Table 2 and Fig. 3). No significant difference was observed among the groups using the ANOVA of PK parameters (*p* > 0.05).

**Table 2:** PK parameters (half-life, AUC, C_max_, and T_max_) and Fc-FcRn affinity parameters of trastuzumab charge variants

**Samples**	**Half-life (h)**	**AUC **_0-28_ (µg*day.mL ^-1^ **)**	**C**_max_ **(µg.mL** ^-1^ **)**	**T**_max_ **(min)**	**Fc-FcRn affinity parameters**			**p** ***I*** ** values**
					**Ka**	**Kd**	**KD**	
APs	47.68 ± 7.24	625.4 ± 53.4	231.37 ± 22.12	5 ± 0	1.886×10^5^	0.16	8.718 × 10^-7^	8.1-8.4
MP	79.47 ± 16.44	953.8 ± 74.3	294.52 ± 25.32	5 ± 0	1.040×10^5^	0.0036	3.488 × 10^-8^	8.5
BPs	74.32 ± 14.53	772.0 ± 54.1	236.77 ± 24.41	5 ± 0	3.790×10^5^	0.2	5.377 × 10^-7^	8.6-8.7

Representative p*I* values were measured by cIEF method. Analysis of PK parameters (HL, AUC, C_max_, T_max_) was performed in triplicate.

## DISCUSSION

Acidic and basic variants are inevitable heterogeneities during the production of mAbs^[^^24^^]^. Many studies have been conducted to elucidate the role of mAbs charge variants in biological activity, binding specificity, and the clearance^[^^4^^,^^12^^,^^13^^,^^25^^,^^26^^]^. Since mAbs charge variants are not similar in their impacts on safety and efficacy^[^^11^^]^, determining these effects is vital to define the design space in the downstream process of each mAb. In this regard, it seems that the charge variant profile of each therapeutic mAb is unique and it is a key factor for the consistency in the production process^[^^27^^]^.

In this work, the APs, BPs, and main peaks of the proposed biosimilar of trastuzumab were isolated by CEX chromatography. SEC, 2-DE, CD, cIEF, and CGE analyses were conducted to detect the differences of these variants and characterize them, physiochemically. As a quality attribute, aggregation likely leads to loss of efficacy and immunogenicity^[^^28^^]^. It was found that charge variants and the MP of trastuzumab possessed a negligible amount of fragments and aggregate levels, which is in contrast to another report implying that the acidic variants of the IgG_1_ had a higher percentage of Fab fractions^[^^29^^]^, and the basic variants of a humanized IgG_1_ were more enriched with aggregates^[^^12^^,^^25^^]^. It is well-understood that charge differences induced by N-terminal modification such as the presence of glutamine or pyroglutamic acid can impair the inter-molecular interaction and cause aggregation^[^^30^^]^. Conformational changes in secondary and tertiary structures can cause biological activity differences^[^^26^^]^. Our CD analysis did not reveal any significant differences in higher-order structure, which is in direct line with Tang et al.’s study^[^^31^^]^.

Each of the isolated charge variants was used in the *in vitro* studies including affinity, efficacy, and both in vitro and in vivo PK) . The SPR analysis showed that the binding affinity of these variants towards HER2 was comparable to each other (Supplementary Table 2). However, Dakshinamurthy et al.^[^^25^^]^ reported the decreased binding of acidic variants of another biosimilar of trastuzumab to HER2. Indeed, changes are mostly significant when happening in the CDRs and when affecting antigen binding. CDRs in antibodies are vulnerable to deamidation events such as succinimide formation, which can reduce mAb antigen-binding affinity^[^^32^^]^. Based on Vlasak et al.^[^^33^^]^, the deamidation of asparagine in the CDR1 of a humanized IgG1 resulted in the decreased binding to its antigen. Another report showed that the deamidation of CDR2 in an unspecified mAb resulted in a 14-fold reduction in antigen binding^[^^34^^]^. Bults et al.^[^^35^^]^ exhibited that the *in* *vivo* deamidation of CDRs of trastuzumab decreased the binding of trastuzumab to HER2 receptor. Also, in agreement with our findings, some reports showed no significant variation in terms of HER2 binding was seen among trastuzumab charge variants^[^^7^^,^^36^^]^. The disagreement between different reports might be related to varying molecular changes in the charge variants, which originate from the process parameters and could be changed during biosimilar production processes. According to our *in vitro* results, modification caused minor differences in the molecule surface charge that may not occur in the antigen-binding site.

In the current study, the difference in binding affinity of charge variants towards FcRIIIa and FcRIIIb was also comparable (Supplementary Table 2). Hintersteiner et al.^[^^10^^]^ demonstrated that the basic variants of a chimeric anti-GD2 antibody shoed an increased binding to FcRIIIa receptor. It seems that the location and the modification level are crucial to the final effect on the binding affinity^[^^24^^]^. Hintersteiner and colleagues^[^^10^^]^ suggested that the main form of a chimeric anti-GD2 antibody has more affinity to FcRn^[^^10^^]^, while the present* in vitro* PK evaluation by SPR demonstrated no significant difference among the main, acidic and basic variants of trastuzumab (Table 2). Additionally, acidic and basic variants did not statistically show significant responses in the *in vivo* PK assay (Table 2), which was confirmed by other reports on different mAbs^[^^12^^,^^13^^,^^25^^,^^37^^]^. According to the results of our cIEF test, the difference in the p*I* of the acidic, basic and main groups of the proposed mAb was less than one unit (Table 2). Certain reports have also suggested that a change of about one or more units in the p*I* of charge variants could affect the mAbs pharmacokinetics^[^^16^^,^^38^^]^. The mAbs with higher p*I* values exhibited faster clearance compared to the ones with lower p*I *^[^^39^^]^. 

Based on the findings of this study, the charge variants of a trastuzumab biosimilar had no remarkable influence on their binding affinity to HER2, FcRIIIa, FcRIIIb, and FcRn. Besides, the *in vivo* PK parameters of these variants showed no significant difference among PK parameters of charge variants. A large number of studies regarding the effect of mAb charge variants on binding affinity and PK parameters indicate that the nature and composition of acidic and basic variants could be highly critical in determining their influence on efficacy and safety. The nature and composition of charge variants are dependent on mAb types and the production process. Therefore, the comparability study of charge variant profiles during the development and production of a biosimilar mAbs is essential for biosimilar safety and efficacy. 

## DECLARATIONS

### Acknowledgments

We would like to thank AryoGen Pharmed (Karaj, Iran) for providing us with the proposed biosimilar of trastuzumab.

### Ethical statement

The study protocol was approved by the Ethics Committee of Pasteur Institute of Iran, Tehran (ethical code: IR.PII.REC.1396.38). All procedures were performed in accordance with the ethical Helsinki standards. All the authors have read and approved the contents of the final manuscript and agreed to publicize this manuscript.

### Data availability

The analyzed data sets generated during the study are available from the corresponding author on reasonable request.

### Author contributions

FT: designed the project and experiments, wrote the manuscript and supervised analyses; MM: performed experiments and data analysis, performed *in vivo* experiments, performed capillary electrophoresis experiments and wrote the manuscript; MRG: performed experiments and data analysis; PJ: performed experiments and data analysis; SM: performed *in vivo* experiments; BA: performed capillary electrophoresis experiments; SS: performed CD experiments and analysis; BV: designed the project and experiments, wrote the manuscript and supervised analyses.

### Conflict of interest

We have no conflicts of interest to disclose.

### Funding/support

This work was supported by Pasteur Institute of Iran (grant no. 1004).

## Supplementary Materials


