# Supplementary material for: Trastuzumab Charge Variants: a Study on Physicochemical and Pharmacokinetic Properties
Source: Iran Biomed J. 2023 Jan 1;27(2-3):108–16. doi: 10.52547/ibj.3837 (PMC10314757; doi:10.52547/ibj.3837)
Supplement: Supplementary file 1 [file ibj-27-108-s001.pdf]

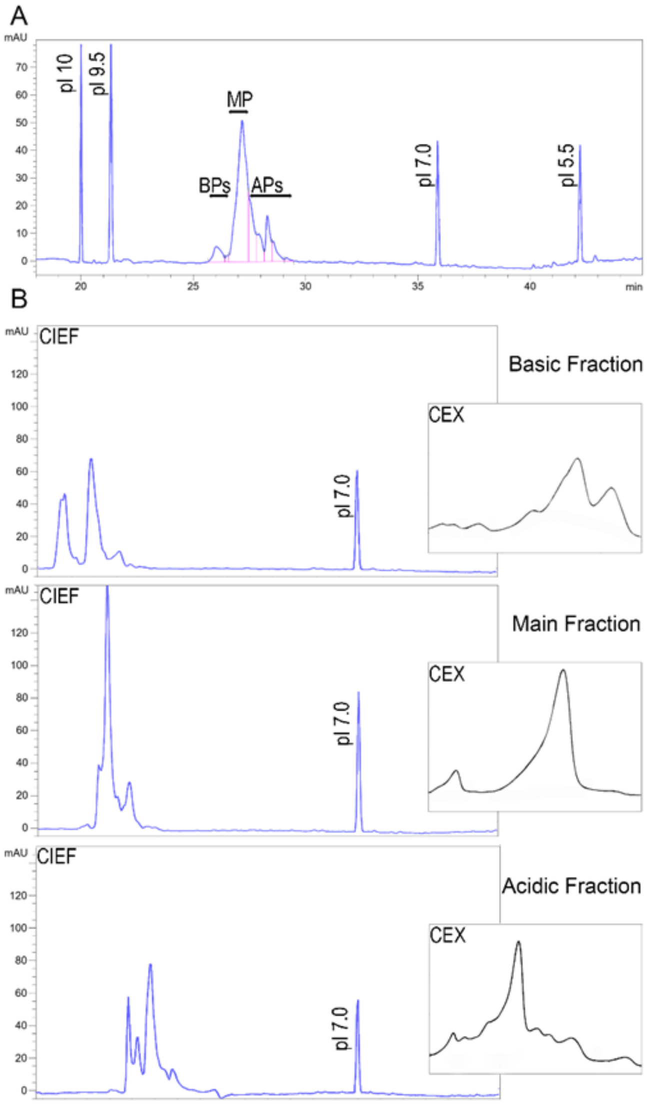

Supplementary Fig. 1. cIEF of trastuzumab and its pooled charge variants fractions. (A) Trastuzumab capillary isoelectric focusing (cIEF) charge variant pattern; (B) comparison of electropherograms of charge variant fractions with each other and with the related cation exchange chromatography (CEX) chromatograms. The migration pattern of charge variants in cIEF is the opposite of their pattern in CEX.

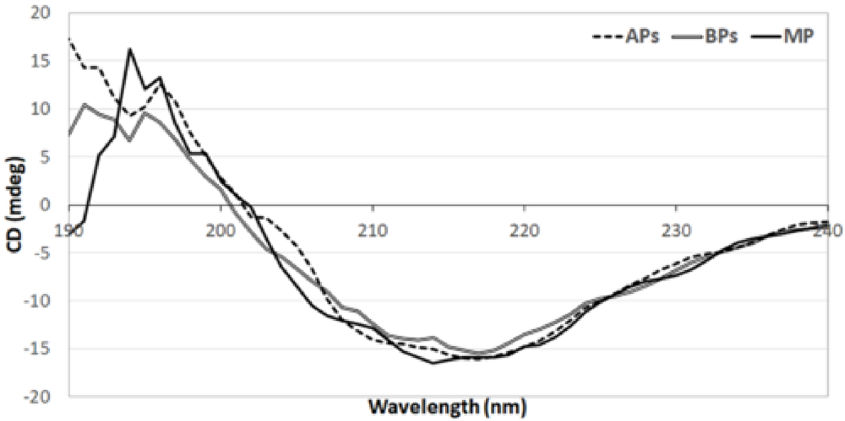

Supplementary Fig. 2. Far-UV circular dichroism spectra of trastuzumab charge variant fractions recorded between 190-250 nm. AP, acidic peak; BP, basic peak; MP, main peak

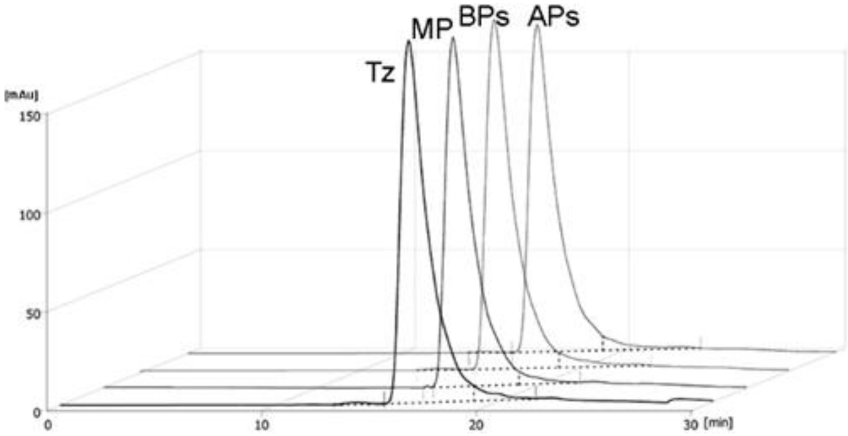

Supplementary Fig. 3. Size exclusion chromatography analysis of trastuzumab and its charge variants. Tz, trastuzumab sample before fractionation; AP, acidic peak; BP, basic peak; MP, main peak

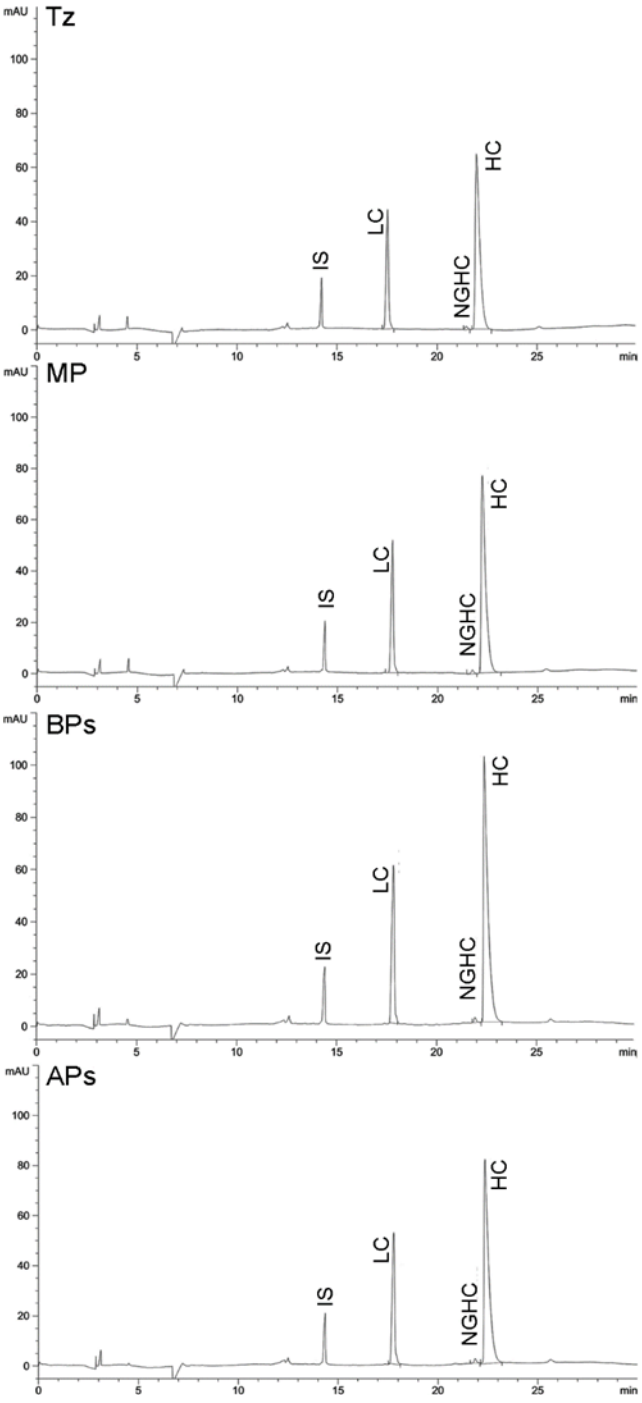

Supplementary Fig. 4. The reduced capillary electrophoresis-sodium dodecyl sulfate of trastuzumab and its charge variants. Tz, trastuzumab sample prior to fractionation; IS, internal standard; LC, light chain; NGHC, non-glycosylated heavy chain; HC, heavy chain

Supplementary Table 1. Percentage of different conformations of trastuzumab charge variants obtained by CD analysis

Variants	Helix (%)	Beta (%)	Turn (%)	Random (%)
Acidic peak	26.8	30.4	14.2	28.6
Main peak	19.4	40.2	15.5	24.9
Basic peak	23.2	30.4	16.1	30.3

Supplementary Table 2. The Ka, Kd, and KD kinetic parameters for the affinity of the acidic, basic, and main form of trastuzumab towards FcγRs and HER2 antigen obtained by surface plasmon resonance

Receptors	Acidic peak	Main peak	Basic peak	Affinity parameters
HER2	9.37×10^{5}	2.97×10^{4}	5.77×10^{4}	
FcγRIIIa	1.02×10^{5}	3.66×10^{4}	3.13×10^{4}	Ka ($M^{-1}S^{-1}$)
FcγRIIIb	1.06×10^{5}	1.54×10^{4}	2.56×10^{4}	
HER2	3.36×10^{-4}	2.07×10^{-4}	2.95×10^{-4}	
FcγRIIIa	0.08	0.09	0.05	Kd (S^{-1})
FcγRIIIb	0.82	0.34	0.33	
HER2	3.59×10^{-9}	2.03×10^{-9}	2.78×10^{-9}	
FcγRIIIa	2.65×10^{-6}	2.54×10^{-6}	3.34×10^{-6}	KD (M)
FcγRIIIb	1.43×10^{-5}	1.09×10^{-5}	1.29×10^{-5}	
